# Factor Analysis of Low-Frequency Repetitive Transcranial Magnetic Stimulation to the Temporoparietal Junction for Tinnitus

**DOI:** 10.1155/2016/2814056

**Published:** 2016-10-26

**Authors:** Hui Wang, Bei Li, Meiye Wang, Ming Li, Dongzhen Yu, Haibo Shi, Shankai Yin

**Affiliations:** ^1^Department of Otolaryngology Head and Neck Surgery, Shanghai Jiao Tong University Affiliated Sixth People's Hospital, Shanghai 200233, China; ^2^Department of Otolaryngology, Tinnitus and Hyperacusis Center Yueyang Hospital of Integrated Traditional Chinese and Western Medicine Affiliated to Shanghai University of Traditional Chinese Medicine, Shanghai 200437, China

## Abstract

*Objectives*. We investigated factors that contribute to suppression of tinnitus after repetitive transcranial magnetic stimulation (rTMS).* Methods*. A total of 289 patients with tinnitus underwent active 1 Hz rTMS in the left temporoparietal region. A visual analog scale (VAS) was used to assess tinnitus loudness. All participants were interviewed regarding age, gender, tinnitus duration, laterality and pitch, audiometric parameters, sleep, and so forth. The resting motor thresholds (RMTs) were measured in all patients and 30 age- and gender-matched volunteers.* Results*. With respect to different factors that contribute to tinnitus suppression, we found improvement in the following domains: shorter duration, normal hearing (OR: 3.25, 95%CI: 2.01–5.27, *p* = 0.001), and without sleep disturbance (OR: 2.51, 95%CI: 1.56–4.1, *p* = 0.005) adjusted for age and gender. The patients with tinnitus lasting less than 1 year were more likely to show suppression of tinnitus (OR: 2.77, 95%CI: 1.48–5.19, *p* = 0.002) compared to those with tinnitus lasting more than 5 years. Tinnitus patients had significantly lower RMTs compared with healthy volunteers.* Conclusion*. Active low-frequency rTMS results in a significant reduction in the loudness of tinnitus. Significant tinnitus suppression was shown in subjects with shorter tinnitus duration, with normal hearing, and without sleep disturbance.

## 1. Introduction

Tinnitus is a subjective, mostly transitory, phantom auditory perception of sound that affects millions of people at some point in their lives [1]. It is estimated that tinnitus will become chronic and severely affect the quality of life in 1%–3% of the general population [2]. About 20% of adults that experience tinnitus will require clinical intervention [3]. Tinnitus can occur constantly or intermittently in one or both ears or centrally in the head and can be perceived as coming from within the head. There is convincing evidence from functional imaging and neurophysiological studies that central mechanisms are responsible for most cases of tinnitus [4–6], which may be caused by (1) changes in the firing pattern of neurons in the central auditory system, (2) changes in burst firing and neural synchrony, and (3) cortical tonotopic map reorganization [7]. All of the above may occur due to alterations in neuronal activity in the brain cortex, which suggests promising treatment strategies for tinnitus.

Based on the above findings, repetitive transcranial magnetic stimulation (rTMS) of the temporal and temporoparietal cortex has been proposed as a promising treatment for chronic tinnitus. Its mechanism of action involves targeting the hyperactivity/abnormal synchronization within the auditory cortex to suppress tinnitus [8–10]. rTMS temporarily disrupts a circumspect area of the cortex that interrupts normal functioning and has acute and chronic effects in tinnitus patients. Several clinical studies consistently showed a reduction of tinnitus severity after application of rTMS to the left temporoparietal region of the cortex [11–13]. However, the treatment results showed high interindividual variability [14, 15], indicating the need for optimization of the indications. This variability also indicates that a large sample study is needed.

The present study was using active rTMS performed to investigate optimization of the indications by evaluating the different factors that contribute to tinnitus suppression after low-frequency rTMS for the treatment of chronic tinnitus.

## 2. Methods

### 2.1. Subjects

Patients suffering from nonpulsatile and constant tinnitus examined and treated at the Affiliated Sixth People's Hospital Otolaryngology Department, Shanghai Jiao Tong University, between December 2014 and December 2015, were included in this study. With local ethics committee approval, written informed consent was obtained from all subjects prior to enrollment, and the possible consequences of the study were explained. Our registration number is ChiCTR-INR-16008092. Upon recruitment, the subjective tinnitus loudness perception in patients was determined using a visual analog scale (VAS) ranging from 0 to 10, where 0 indicates no tinnitus and 10 indicates the worst possible tinnitus-related discomfort. VAS improvement in responders decreased to 20% of the basal score indicating no reduction, 40%, 60%, and 80% of the basal score indicating slight, marked, and strong reduction, and 100% reduction indicating complete suppression of tinnitus immediately after stimulation. The reduction range from 40% to 100% is considered as “good effect.” Measurements were made before, immediately, and 2 weeks after the last intervention session.

Detailed histories were obtained from all patients, including a clinical examination and audiogram. Hearing level was assessed by using an audiometer in a soundproof room, and the loudness and pitch of tinnitus were evaluated with a TinniTest audiometer. Normal hearing was defined as normal audiogram (threshold < 25 dB HL at all frequencies from 0.25 to 8 kHz) as well as a normal tympanometric curve of type A. The ipsilateral and contralateral stapedial reflexes were also included. All participants were comprehensively interviewed for information regarding their age, gender, tinnitus duration, tinnitus laterality, pitch of tinnitus, sleep quality, accompanying symptoms, and so forth.

### 2.2. rTMS Procedure

All of the patients underwent rTMS over the left temporoparietal cortex region. As described in our previous study [6], rTMS consisted of 1000 stimuli at 1 Hz daily and 110% of the motor cortex threshold for 5 consecutive days per week (Monday to Friday) for 2 weeks. For repetitive pulses, TMS was delivered through a focal figure-eight magnetic coil connected to a magnetic stimulator (MagPro R30; MagVenture, Farum, Denmark). The resting motor threshold (RMT) was defined as the lowest stimulator output intensity capable of inducing motor evoked potentials (MEPs) of at least 50 *μ*V peak-to-peak amplitude in the relaxed state in at least 5 of 10 consecutive trials. Thirty volunteers with normal hearing served as controls, whose RMTs were obtained. The tinnitus subjects and controls were age- and gender-matched.

### 2.3. Statistical Analysis

All statistical analyses were performed using SPSS v 16.0 (SPSS, Chicago, IL, USA). Prior to analysis, all variables were examined for their normality. All categorical variables were analyzed by the Chi-square test. We calculated the odds ratio (OR) and associated 95% confidence interval (CI) for demographic factors using the Mantel–Haenszel method. Logistic regression was performed to identify the factors contributing to tinnitus suppression after the intervention. In all analyses, *p* < 0.05 was taken to indicate statistical significance.

## 3. Results

The stimulation protocols were well tolerated, and all the patients completed the treatment except three patients who reported transient mild to moderate headache, two patients who reported transient worsening of their tinnitus, and two patients that complained of vertigo. None of the patients developed seizures or other serious side effects or adverse effects. A total of 289 patients with chronic unilateral or bilateral tinnitus (137 male, 182 female, age range: 19–87 years, mean 57 ± 14.7 years) were included in the study. In the active stimulation group, 76 (26.3%) patients reported purely left-sided tinnitus, 50 (17.3%) patients reported purely right-sided tinnitus, 129 (44.6%) patients described their tinnitus as bilateral, and 34 (11.8%) patients described their tinnitus as originating within the head. The duration of tinnitus ranged from 7 months to 40 years, with a median of 5.8 years, and approximately 15.2% of the subjects had experienced tinnitus for more than 5 years. The patients rated their tinnitus loudness on the VAS, and the median score was 6.5 (range, 4–10). A total of 169 (58.5%) of the 289 patients reported tonal tinnitus and 108 (37.4%) patients described noisiform tinnitus. Twelve (4.2%) patients could not determine the pitch of tinnitus or their pitch of tinnitus was unmatchable. Audiometric assessment showed normal hearing in 143 of 289 subjects (49.5%) and hearing loss in 146 (50.5%). Tinnitus interfered with sleep in 129 of the 289 patients.

The clinical characteristics of the tinnitus patients are shown in Table 1. Participants completed a questionnaire assessment of medical history before and immediately after the last intervention. Information on age, gender, tinnitus laterality, duration of tinnitus, hearing level, pitch of tinnitus, accompanying symptoms, underlying diseases, and sleep quality was included in the analyses.

rTMS showed a good effect in 138 of the patients included in the study (47.8%) and no effect in 151 patients (52.2%) in the active group. With respect to different factors contributing to tinnitus suppression, we observed improvement after active rTMS in the following domains: age, gender, duration of tinnitus, tinnitus laterality, audiometric parameters, and sleep. Significant tinnitus suppression was associated with younger age, male gender, shorter duration of tinnitus, tinnitus located centrally in the head, normal hearing, and no sleep disturbance at night (*p* < 0.05). These patients showed no significant differences in pitch of tinnitus, accompanying symptoms, such as headache and vertigo, and underlying diseases, such as hypertension, diabetes, and heart disease (*p* > 0.05) (Table 1).

Before the intervention, the average baseline VAS score in patients with active stimulation was 5.5. The score was decreased to 2.7 immediately after the last active intervention. After active rTMS, 51.3% (SD = 20.6%) patients experienced a significant reduction in tinnitus loudness, as evidenced by VAS scale.

In the present study, the duration of tinnitus was correlated with tinnitus suppression by rTMS. The intervention was more effective in suppressing tinnitus in patients that had suffered from tinnitus for only a short time. Multinomial logistic regression analysis was performed with tinnitus duration as a dependent variable and the change in VAS score before and after stimulation as a predictor variable. There was a linear (negative) correlation between the duration of tinnitus and the degree of tinnitus suppression by rTMS (*p* = 0.013). Patients with tinnitus lasting less than 1 year were more likely to show suppression of tinnitus (OR: 2.77, 95% CI: 1.48–5.19, *p* = 0.002) immediately after the last intervention compared to those with tinnitus lasting more than 5 years (Table 2). Tinnitus could be suppressed, on average, in 60.2% of patients with tinnitus lasting less than 1 year but only in 51.8% of those with symptom duration of less than 2 years and only in 40.7% of those with symptoms lasting for more than 2 years.

When compared to patients with sensorineural hearing loss, those with normal hearing were more likely to show suppression of tinnitus (OR: 3.25, 95% CI: 2.01–5.27, *p* = 0.001) immediately after the last intervention. In addition, the rTMS treatment showed a significant effect in patients that reported no sleep disturbance (OR: 2.51, 95% CI: 1.56–4.10, *p* = 0.005). Patients with tinnitus located centrally in the head often reported a reduction in tinnitus after the rTMS procedure in contrast to those with left or right tinnitus (OR: 1.63, 95% CI: 0.62–4.31 *p* = 0.324). Although rTMS was only applied over the left temporoparietal cortex, tinnitus was decreased equally well regardless of whether it was predominantly experienced in the left or right ear. There was no significant difference in the outcome between ipsilateral and contralateral rTMS. There was no significant difference in the outcome between bilateral and left-sided stimulation either (Table 2).

**Figure 1:**
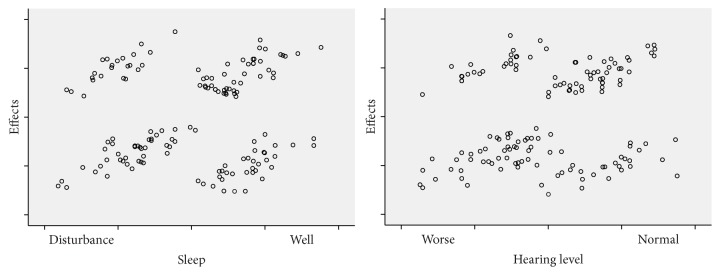
Correlations were observed between sleep and hearing level and efficacy of rTMS. The intervention was more likely to suppress tinnitus in subjects with normal hearing and those without sleep disturbance.

To investigate the major determinants of the effects of rTMS on tinnitus, we performed stepwise linear regression analyses between baseline characteristics, such as age, sex, duration of tinnitus, tinnitus laterality, hearing level, sleep quality, and efficacy of treatment. Age, gender, duration of tinnitus, hearing level, and sleep quality showed significant associations with the efficacy of rTMS, while tinnitus laterality showed no considerable effect (*p* = 0.233). On multiple regression analysis adjusted for age and gender, the patients with shorter tinnitus duration and normal hearing and without sleep disturbance showed maximal induction of tinnitus suppression by rTMS (Figure 1).

**Figure 2:**
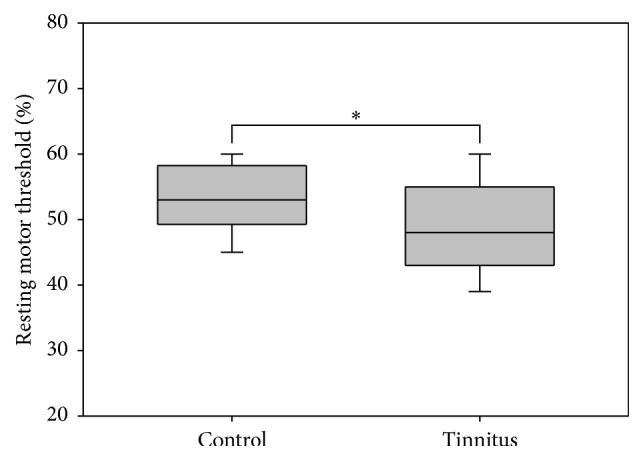
The resting motor threshold was significantly lower in tinnitus patients compared with healthy volunteers.

Baseline RMT in tinnitus patients was 48.5%, and that in healthy controls was 53.1%, indicating that tinnitus patients had significantly lower RMTs (Figure 2) than healthy volunteers (*t* = 2.926, *p* = 0.004).

## 4. Discussion

The present study demonstrated that active low-frequency rTMS results in a significant decrease in the loudness of tinnitus. With respect to different factors that contribute to tinnitus suppression, active rTMS induces maximal tinnitus suppression in subjects with shorter tinnitus duration, normal hearing, and without sleep disturbance. The tinnitus patients have enhanced brain excitability compared with the controls.

The use of rTMS in the treatment of tinnitus stems from the development of models of central generation induced by auditory deafferentation (neural plasticity with hypersynchrony or hyperactivity of cortical and subcortical auditory and nonauditory areas) [16–19]. The results of the present study showed that tinnitus can be transiently suppressed partially or completely by rTMS in approximately 47.8% of tinnitus patients. Tinnitus duration, hearing level, and sleep quality were identified as positive predictors, in agreement with previous studies [20]. The amount of tinnitus suppression by rTMS was inversely correlated with the duration of symptoms; that is, shorter duration of tinnitus was associated with a greater suppressive effect. Two years seems to be an important turning point for obtaining a beneficial outcome. Tinnitus lasting less than 1 year could be suppressed on average in 60.2% of responding patients, decreasing to 51.8% of those with a symptom duration of less than 2 years and 40.7% after more than 2 years. It is possible that the central network involved in tinnitus becomes less plastic and less responsive to rTMS intervention over time. In addition, the degree of tinnitus suppression achieved through rTMS depended on the hearing of the patients. Hearing impairment was also identified as a negative predictor in our study. When compared to patients with sensorineural hearing loss, those with normal hearing were more likely to show suppression of tinnitus immediately after the last intervention. These observations suggested that hearing loss may represent an ongoing trigger for the generation of tinnitus and both reduce and shorten the TMS treatment effects. In addition, the deprivation of auditory input may lead to disinhibition in the central auditory system, which in turn may exacerbate the plastic changes in neural functioning that could underlie tinnitus [21]. In addition, the reduction of inhibition in central auditory structures leads to hyperexcitability of circumscribed regions of the central auditory system, as evidenced by the enhanced brain excitability indicated in the present study. Moreover, rTMS treatment had a significant effect in patients without sleep disturbance. Nonauditory areas, such as the frontoparietal areas and limbic areas, have been suggested to be involved in the pathophysiology of tinnitus, as evidenced by the functional imaging data in our previous study [6]. These interactions between auditory and nonauditory brain regions may explain why tinnitus is perceived as bothersome. These mechanisms can lead to comorbid conditions, such as concentration problems, depression, and sleep disturbances [22]. Accordingly, impaired sleep quality shows an increased prevalence in cases of chronic tinnitus. In addition, patients frequently report that tinnitus prevents them from falling asleep.

Low-frequency rTMS, which is known to suppress cortical excitability, has been used successfully to interfere with neural functioning in the temporoparietal cortical region as the magnetic field passes through the skull and induces a small secondary current in the cortex [9, 23]. Accordingly, low-frequency rTMS has been used to treat tinnitus [24, 25]. In addition, TMS can also be used as a diagnostic tool for the assessment of motor cortex excitability by quantifying contractions of peripheral muscles induced by stimulation of the corresponding motor cortex representation. In the present study, tinnitus patients were shown to have enhanced brain excitability compared with controls, which was consistent with previous studies suggesting that central mechanisms are responsible for at least some cases of tinnitus [26]. The enhanced brain excitability was accompanied by a decrease in glucose metabolism and inhibitory-acting *γ*-aminobutyric acid (GABA) in the stimulated temporal cortex and an increase in cingulate and frontal areas but also in motor cortex, as evidenced by the previous study [27–29]. Many efforts have been made to gain insight into the neurophysiological mechanisms of tinnitus [30]. This knowledge can contribute to investigations into the pathophysiology of tinnitus.

Evidence from previous studies suggested that a functional network of several cortical areas may be responsible for tinnitus, but the precise region affected remains unclear [31, 32]. Using PET imaging, Plewnia et al. [33] found greater activity in the left auditory cortex of chronic tinnitus patients, regardless of the side of symptoms, or centrally within the head. Although rTMS was only applied over the left temporoparietal cortex in the present study, patients responded equally well regardless of whether their symptoms were predominantly in the left or right ear. The rate of response in tinnitus located centrally in the head was even higher than that for tinnitus located predominantly in the ears. However, on multiple regression analysis adjusted for age and gender, tinnitus laterality had no considerable effect.

In conclusion, active low-frequency rTMS resulted in significant suppression of the loudness of tinnitus, especially in patients with a shorter tinnitus duration and normal hearing and without sleep disturbance. Tinnitus patients showed enhanced brain excitability compared with controls. Future studies may significantly benefit from emerging imaging techniques to identify the mechanisms underlying tinnitus and from stimulation protocols that will together determine the optimal site for targeting rTMS stimulation.

## Figures and Tables

**Table 1:** Clinical characteristics of the patients suffering from tinnitus.

Independent variables	Active group		*N*	*p* value
	No effect%	Improved%		
*Age (yrs)*				
<30	5 (29.41)	12 (70.59)	17	0.012
31–50	30 (48.39)	32 (51.61)	62	
51–70	88 (51.16)	84 (48.84)	172	
>70	28 (73.68)	10 (26.32)	38	
*Gender*				
Male	56 (45.16)	68 (54.84)	124	0.043
Female	95 (57.58)	70 (42.42)	165	
*Underlying diseases *				
Good condition	121 (50.21)	120 (49.79)	241	0.154
Underlying diseases	30 (62.50)	18 (37.50)	48	
*Accompanied symptoms*				
No accompanied symptoms	95 (47.74)	104 (52.26)	199	0.052
Headache	18 (69.23)	8 (30.77)	26	
Dizziness	38 (59.38)	26 (40.62)	64	
*Pitch of tinnitus*				
Tone	79 (46.75)	90 (53.25)	169	0.06
Noise	66 (61.11)	42 (38.89)	108	
Uncertainty	6 (50.00)	6 (50.00)	12	
*Tinnitus laterality*				
Left ear	38 (50.00)	38 (50.00)	76	0.025
Right ear	28 (56.00)	22 (44.00)	50	
Bilateral	75 (58.14)	54 (41.86)	129	
Ringing in the head	10 (29.41)	24 (70.59)	34	
*Audiometric parameters*				
Normal hearing	53 (37.59)	88 (62.41)	141	0.001
Sensorineural hearing loss	98 (66.22)	50 (33.78)	148	
*Tinnitus duration*				
≤1 yr	29 (39.73)	44 (60.27)	73	0.013
1 yr < *x* ≤ 2 yrs	26 (48.15)	28 (51.85)	54	
2 yrs < *x* ≤ 5 yrs	62 (64.58)	34 (35.42)	96	
>5 yrs	34 (51.51)	32 (48.49)	66	
*Sleep*				
Sleep disturbance	84 (64.62)	46 (35.38)	130	0.005
Sleep well	67 (42.14)	92 (57.86)	159	

**Table 2:** Multivariate stepwise logistic regression analyses models for different factors that contribute to tinnitus suppression.

Independent variables	Odds ratio	95%	*p* value
		Confidence interval	
*Age (yrs)*			
<30	0.24	0.06–1.07	*p* = 0.061
31–50	0.48	0.14–1.69	*p* = 0.255
51–70	0.29	0.08–1.12	*p* = 0.072
>70	1		
*Gender*			
Female	1		
Male	1.57	0.88–2.76	*p* = 0.122
*Tinnitus laterality*			
Left ear	1		
Right ear	0.81	0.36–1.81	*p* = 0.602
Bilateral	0.85	0.45–1.60	*p* = 0.613
Ringing in the head	1.63	0.62–4.31	*p* = 0.324
*Hearing level*			
Normal hearing	3.25	2.01–5.27	*p* = 0.001
Sensorineural hearing loss	1		
*Duration of tinnitus*			
>5 yrs	1		
2 yrs < *x* ≤ 5 yrs	1.52	0.70–3.10	*p* = 0.441
1 yr < *x* ≤ 2 yrs	1.61	0.82–3.20	*p* = 0.731
≤1 yrs	2.77	1.48–5.19	*p* = 0.002
*Sleep*			
Sleep disturbance	1		
Without sleep disturbance	2.51	1.56–4.10	*p* = 0.005
